# Incidence of ‘Low-Risk but Not No-Risk’ Features of Cancer Prior to High-Risk Feature Occurrence: An Observational Cohort Study in Primary Care

**DOI:** 10.3390/cancers15153936

**Published:** 2023-08-02

**Authors:** Sarah F. Moore, Sarah J. Price, Jennifer Bostock, Richard D. Neal, Willie Hamilton

**Affiliations:** 1 Faculty of Health and Life Sciences, University of Exeter, Exeter EX2 4TH, UK; 2 Policy Research Unit in Cancer Awareness, Screening and Early Diagnosis, Queen Mary University of London, Mile End Rd., London E1 4NS, UK

**Keywords:** cancer, early diagnosis, referrals, primary care

## Abstract

**Simple Summary:**

The earlier we can find cancers, the better the chance of survival. Currently, when a patient visits their General Practitioner (GP) with a symptom that might be cancer, they are referred to the hospital for urgent further testing if the symptom is a high risk for cancer. This study aims to find out whether patients who see their GP with a high-risk symptom have also seen their GP with a ‘low-risk but not no-risk’ symptom in the previous year. If this is the case, lowering the referral threshold might allow them to be identified earlier, resulting in improved outcomes if diagnosed with cancer.

**Abstract:**

Diagnosing cancer may be expedited by decreasing referral risk threshold. Clinical Practice Research Datalink participants (≥40 years) had a positive predictive value (PPV) ≥3% feature for breast, lung, colorectal, oesophagogastric, pancreatic, renal, bladder, prostatic, ovarian, endometrial or laryngeal cancer in 2016. The numbers of participants with features representing a 1–1.99% or 2–2.99% PPV for same cancer in the previous year were reported, alongside the time difference between meeting the ≥3% criteria and the lower threshold criteria. A total of 8616 participants had a PPV ≥3% feature, of whom 365 (4.2%) and 1147 (13.3%), respectively, met 2–2.99% and 1–1.99% criteria in the preceding year. The median time difference was 131 days (Interquartile Range (IQR) 27 to 256) for the 2–2.99% band and 179 days (IQR 58 to 289) for the 1–1.99% band. Results were heterogeneous across cancer sites. For some cancers, participants may progress from presenting lower- to higher-risk features before meeting urgent referral criteria; however, this was not usually the case. The details of specific features across multiple cancer sites will allow for a tailored approach to future reductions in referral thresholds, potentially improving the efficiency of urgent cancer referrals for the benefit both of individuals and the National Health Service (NHS).

## 1. Introduction

The NHS England long-term plan has an explicit target of improving the percentage of patients diagnosed with early-stage cancer from a current level of 55% to 75% in 2028 [1]. One way to achieve this is to increase the number of patients diagnosed through the urgent cancer referral pathway, the use of which has been shown to have higher rates of early diagnosis [2]. At present, General Practitioners (GPs) are encouraged to consider using this urgent referral pathway for patients presenting with features (consisting of symptoms, signs, or test results in isolation or in combination) that have a positive predictive value (PPV) of ≥3% for a subsequent diagnosis of cancer. Our previous study showed that reduction of this threshold to PPV ≥ 2% or ≥1% increased the numbers of patients eligible for urgent referral by 8% and 136%, respectively, across the 11 cancer sites studied (breast, lung, colorectal, oesophagogastric, pancreatic, renal, bladder, prostatic, ovarian, endometrial and laryngeal), though with significant heterogeneity between cancer sites [3].

The classic medical teaching for solid tumor development is that of local to regional to metastatic spread [4,5]. Intuitively, therefore, physicians often think that the same must be true of symptom development and that patients with cancer develop features in a step-wise manner, beginning with low-risk features, which subsequently develop into high-risk features associated with regional or metastatic spread. However, there is limited evidence for this [6] and also increasing evidence to the contrary, with patients skipping classic stages possibly driven by early micro-metastases [7].

If the symptom progression hypothesis is true, or even partially true, then by reducing the thresholds for urgent referral, we should not only increase the number of patients eligible for referral but also be able to identify them earlier. One way to investigate this is to identify patients who present with a higher-risk feature and then to look back to ascertain if lower-risk features occurred in the previous year. 

If this shows that patients with high-risk symptoms (PPV ≥ 3%) develop ‘low-risk but not no-risk’ symptoms (PPV = 1 to 1.99% or 2 to 2.99%) prior to the high-risk symptoms, that could help strengthen the case for reducing urgent cancer referral thresholds. It would give us evidence for the symptom progression model and might allow us to identify these patients earlier in their disease when their cancers are less advanced. It could also lead to a tailored approach to any future reduction in referral thresholds by cancer site, potentially improving the efficiency of urgent cancer referrals for the benefit both of individuals and the NHS. 

## 2. Materials and Methods

This study builds on the research conducted by Moore et al. [3], where a more detailed explanation of the original methods can be found. They are summarised here with more detail on the key differences in this project.

### 2.1. Study Design, Setting, and Sample Selection

This was an observational, descriptive, cross-sectional study set in primary care using data from the Clinical Research Practice Datalink (CPRD). The initial population was a random sample of 150,921 patients resident in England, aged 40 or over, and with continuous registration at a CPRD general practice before 1 January 2016 was studied. The study period included 2 years, from 1 January 2015 to 31 December 2016, to allow full capture of patients meeting criteria for multiple or recurrent symptoms in 2016. The sample size was determined for the original study; see Moore et al. [3]. 

The cancers studied were the 11 commonest internal cancers: bladder, breast, colon or rectum, endometrium, kidney, larynx, lung, oesophagus or stomach, ovary, pancreas, and prostate. 

This descriptive study is a secondary analysis of a subpopulation of the original dataset. The new inclusion criterion was a presentation to primary care with a feature with a risk of cancer ≥3% in 2016. 

### 2.2. Identification of Features

A list of the possible features with a risk of cancer ≥ 1% was compiled for each of the 11 cancer types studied using the information from the tables of studies contained in the NICE (National Institute for Clinical Excellence) guidance document for practitioners in England on ‘suspected cancer: recognition and referral’ [8] and a later study for laryngeal cancer, as no primary care data on this cancer site was available at the time of publication [9]. This was published as Appendix A in Moore et al. [3], which also contains information on the specific PPV and study underpinning the data for each feature for each cancer and the population to which it should be applied. For this study, this information was used to create bands of features with 1–1.99% and 2–2.99% PPV (see Appendix A, S4 for the full set of features by cancer site). 

CPRD code lists to define each feature had previously been developed using robust methods [10,11] and were published in Moore et al. Appendix B [3]. We used these code lists to search each participant’s medical record and created individual-level variables for the presence or absence of the feature. Aligned with usual practice, we interpreted the absence of a code as the absence of that feature [12].

### 2.3. Identification of Participants Meeting Criteria

Features by cancer site and by risk band contained in Appendix A (S4) were matched to each participant’s features. For those criteria requiring paired or recurrent features, we required the second feature to occur within a year of the first, in accordance with the methodology underpinning many of the risk estimates [13]. Participants with a history of cancer at the specified site prior to 2016 were excluded from the analysis. 

For each cancer site, if a participant had a feature of that cancer with PPV ≥ 3%, they were assigned an index date corresponding to the first time they met this criterion in 2016. For recurrent and paired features, the date of the second instance or second coded feature was the index date, and this had to occur in 2016. 

In a difference to the previous study, instances of meeting 2–2.99% and 1–1.99% PPV criteria were identified from 1 January 2015 to 31 December 2016 to allow for features occurring up to a year prior to any index date in 2016.

### 2.4. Analysis

Analysis was conducted for each of the cancer sites individually. The date a participant first met a criterion with PPV ≥ 3% risk of cancer in 2016 was identified and nominated as the index date. Next, all instances of participants meeting criteria in the 2–2.99% band from 1 January 2015 to 31 December 2016 were identified, and only those occurring in the 365 days prior to an index date were kept. The earliest of these dates was then chosen to give the best possible chance of early detection and defined as the “2–2.99% date”. The median (Interquartile range (IQR)) difference in days between the index date and the 2–2.99% band date was reported and presented graphically in strip plots. This process was then repeated for the 1–1.99% band of features. The numbers of participants meeting criteria defining the 1–1.99% and 2–2.99% bands in the year before the index date are reported by cancer site, alongside the proportion (with exact binomial 95% confidence intervals (CIs)) of the participants with a PPV ≥ 3% risk of that cancer in 2016. 

### 2.5. Patient and Public Involvement

A member of the Patient and Public Involvement (PPI) group affiliated with the Policy Research Unit (PRU) in cancer awareness, screening, and early diagnosis gave feedback on the proposal for this project and is a co-author of this manuscript. The PRU’s PPI group comprises lay members with an interest in the public, patients and/or carers of those with cancer or suspected cancer and brings the public perspective to our research. 

## 3. Results

### 3.1. Demographics

Our initial sample was the same as that described in our previous study [3] and consisted of 150,921 participants over the age of 40 registered at a practice in England. The database is known to have a slight excess of urban-to-rural practices, but this is unlikely to have introduced bias or reduced generalisability [14]. This study examined the subgroup of this representative sample who met any criteria with PPV ≥ 3% for one of the studied cancers in 2016, as described in Table 1. 

The distribution of the sub-population appears similar to what we might expect, given that in 2016/17, 4162/100,000 females received an urgent cancer referral compared to 2560/100,000 males, and referrals increased with age [15].

### 3.2. Numbers of Patients with Low-Risk Features Prior to High-Risk Features

There were 8616 instances of an individual meeting the ≥3% PPV threshold for urgent cancer referral in 2016, see Table 2. This number is higher than the total number of patients presenting with a PPV ≥ 3% for cancer in Table 1, as some patients met more than one criterion at more than one site and thus appeared more than once in Table 2. 

Of these patients, 365 (4.2%, 95% CI 3.8 to 4.7) presented with a 2–2.99% PPV feature and 1147 (13.3%, CI 12.6 to 14.0) with a 1–1.99% feature of the same cancer site in the preceding year. These results were extremely heterogeneous across cancer sites ranging from 0 (bladder, breast, endometrium, kidney, pancreas) to 9% (larynx) for those meeting 2–2.99% PPV criteria and from 0 (endometrium, larynx, ovary) to 30.7% (oesophagogastric) for those meeting 1–1.99% PPV criteria.

For more details, please see Appendix A S4, which contains tables by cancer site showing the number of patients with features of PPV ≥ 3% and 2–2.99% and PPV ≥ 3% and 1–1.99%. It also details the exact feature pairings between the higher and lower risk features (Appendix A S9, S12, S15, S18, S21, S24, S27, S30, S33, S36, S39).

### 3.3. Length of Time Prior to High-Risk Features That Patients Present with Low-Risk Features

**Figure 1:**
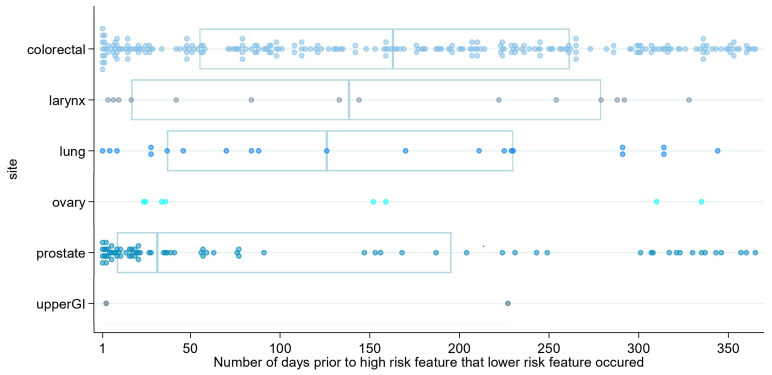
Strip plot to show patients who presented with a ≥3% feature in 2016 also presenting with a feature from a lower band (PPV 2–2.99%) in the preceding year. Note: The *x*-axis shows the number of days prior to the index date that the lower risk feature (2–2.99% PPV band) occurred; each dot represents an individual patient. Only cancer sites for which a lower-risk feature occurred are included in the graph. Boxes show median and interquartile ranges for each cancer site with number of patients >10.

**Figure 2:**
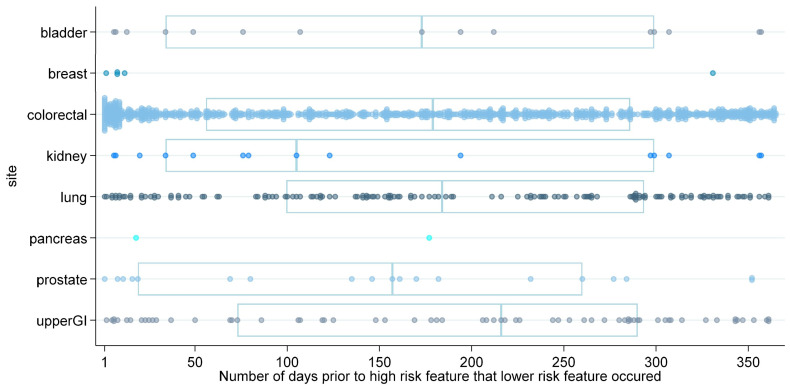
Strip plot to show patients who presented with a ≥3% feature in 2016 also presenting with a feature from a lower band (PPV 1–1.99%) in the preceding year. Note: The *x*-axis shows the number of days prior to the index date that the lower risk feature (1–1.99% PPV band) occurred; each dot represents an individual patient. Only cancer sites for which a lower-risk feature occurred are included in the graph. Boxes show median and interquartile ranges for each cancer site with number of patients >10.

Examining the length of time before high-risk feature presentation for bands PPV 2–2.99% and 1–1.99%, respectively, revealed further heterogeneity, as shown in Figure 1 and Figure 2, respectively. For band 2–2.99%, the median time before ≥3% feature was 131 days (IQR 27 to 256); for band 1–1.99%, the median was 179 (IQR 58 to 289). Figure 1 and Figure 2 show the heterogeneity of this time frame by cancer site, and tables containing further detail by cancer site are contained in Appendix A, S3.

## 4. Discussion

### 4.1. Heterogeneity across Cancer Sites 

The results are extremely heterogeneous depending on the cancer site, as we would expect based on the feature profiles [16]. For 9 of the 11 sites (bladder, breast, colorectal, endometrium, kidney, lung, ovary, pancreas, and oesophagogastric), the reduction of the threshold to ≥2% would help identify fewer than 5% of patients presenting with a higher risk ≥ 3% feature in the preceding year. The only sites where the reduction in the threshold would identify higher proportions of patients earlier are the larynx (9.0%, 5.0 to 14.7), ovary (5.0%, 2.2% to 9.6%), and prostate (7.8%, 6.2% to 9.7%). 

A potential explanation for the higher proportions for prostate and ovary could be that the lower-risk features driving up the numbers are individual versions of paired or recurrent features occurring in the higher-risk category. The increase in the number of patients being identified earlier for prostate appears to rely on the inclusion of features ‘frequency or urgency’ and ‘nocturia’, both of which appear in combination in the higher risk category. The same is true for the ovary and larynx, where ‘abdominal distension’ and ‘hoarseness’ are included at the lower risk threshold as an individual feature and at the higher risk threshold in combination. 

The further reduction of the threshold to ≥1% would identify > 5% of patients for 5 of 11 sites (bladder (6%, CI 3.4 to 9.6%), colorectal (16.7%, CI 15.7 to 17.7%), kidney (5.7%, CI 3.2 to 9.2%), lung (18.1%, CI 15.5 to 20.9%) and oesophago-gastric (30.7%, 24.5 to 37.3)).

Bladder and kidney results are driven by the inclusion of anaemia in men at the lower risk threshold, but absolute numbers are very low. Colorectal shows a high number of patients (878), and the drivers for this are spread across several different lower-risk features but clustered in patients whose PPV ≥ 3% feature is anaemia. Lung also shows a more heterogeneous distribution of feature pairs, though the largest factor is the presence of thrombocytosis as the ≥3% feature. Oesophago-gastric shows the highest percentage of ‘low-risk but not no-risk’ features in the year prior to presentation with a high-risk feature, 86% of which are due to the inclusion of anaemia as a lower-risk feature. This could help improve the selection of patients for urgent referral and potential early diagnosis. 

We know that 41% of patients subsequently diagnosed with cancer have had a common blood test prior to diagnosis, with these figures ranging for a full blood count from 39–69% for the relevant sites in this study (bladder 40%, kidney 46%, lung 39%, stomach 61%, oesophagus 46%, colon 69% and rectum 60%) [17]. This could partially account for these findings, but there is also potential for this to provide further evidence for stratifying patients for early referral based on test results.

The length of time prior to a high-risk feature that a low-risk feature occurs varies by site but for band 2–2.99%, the median time before ≥3% feature was 131 days for band 1–1.99%, the median was 179. This suggests that for the cancer sites where it was possible to calculate this, there could be a benefit in terms of identifying patients significantly earlier in the disease process. This is important as we know identifying patients earlier affects prognosis [18]. Indeed, modelling by Sud et al. suggests that a delay of six months in a colorectal cancer diagnosis (equivalent to the median length of time that lowering thresholds might identify patients earlier in this study) could reduce 10-year survival by 26 to 33%, depending on age [19].

### 4.2. Strengths and Limitations

This study used a large subgroup from a representative sample of patients in England, the target population for the application of NICE guidance [8,12]. The sub-sample was of a distribution similar to that seen in the official urgent cancer referral data for the same year [15]. The slight overrepresentation of women compared with national data is likely due to the inclusion of only 11 solid site tumors, three of which were female-specific and only one male-specific. The code lists to identify possible features of cancer have been developed using robust published methods and used in previous studies [3,10,11]. There are, however, some limitations to CPRD data. If symptoms are recorded in the free text of a consultation or not coded, then they will not be available. This is because CPRD prevents access to free text to protect patient anonymity.

This preponderance of test results could also be accounted for by a differential availability of test results vs. coded symptoms. Analysis of coded data alone underestimates the true number of participants with a symptom by up to 30% for non-site-specific symptoms such as abdominal pain and by 20% for symptoms that are specific and highly predictive of particular cancer [20]. Thus, our estimates of criteria based solely on symptoms, particularly for the 2% and 1% thresholds or for criteria consisting of multiple features, are likely to be moderate underestimates. In contrast, test results are automatically coded into the CPRD record, effectively eliminating missing data for that aspect.

Another key limitation is that this is a secondary analysis of an existing dataset that does not contain follow-up data for these patients to help determine how many of those with the specific combinations of low- and high-risk features went on to develop cancer. This could be addressed in a future study using a larger sample with cancer registry linkage.

### 4.3. Implications for Policy and Practice

Although limited by the lack of linked cancer referral and diagnosis data, the detail of progression (or not) of specific features from low to high-risk across multiple cancer sites will contribute to a tailored approach to any future reduction in referral thresholds. Further work to estimate the potential impact on referrals and diagnoses will strengthen this evidence and increase its potential for influence on policy. Future research could also characterise those with lower-risk features that progress to higher-risk features vs. those that do not. This information could underpin changes to NICE guidance on thresholds for urgent cancer referral in England.

## 5. Conclusions

This study has shown that over 11 cancer sites, the percentage of patients presenting with a ‘low-risk but not no risk’ feature in the year prior to a presentation with a high-risk feature is small (4.2%, 95%CI 3.8% to 4.7%, for 2–2.99% PPV and 13.3%, 95%CI 12.6% to 14.0% for 1–1.99% PPV features).

## Figures and Tables

**Table 1:** Sample demographics.

	Whole Sample			Patients Presenting with Features with PPV ≥ 3% for Cancer in 2016		
Age Category (Years)	Male (n (%))	Female (n (%))	Total	Male (n (%))	Female (n (%))	Total
40–49	19,900 (50.6)	19,434 (49.4)	39,334	173 (13.9)	1071 (86.1)	1244
50–59	20,003 (51.1)	19,149 (48.9)	39,152	342 (25.2)	1014 (74.8)	1356
60–69	15,584 (49.5)	15,924 (50.5)	31,508	575 (37.6)	954 (62.4)	1529
70–79	11,020 (47.5)	12,177 (52.5)	23,917	561 (35.4)	1024 (64.6)	1585
≥80	7136 (40.2)	10,594 (59.8)	17,730	432 (31.0)	962 (69.0)	1394
**Total**	**73,643 (48.8)**	**77,278 (51.2)**	**150,921**	**2083 (29.3)**	**5025 (70.7)**	**7108**

**Table 2:** Number and percentage of patients with a PPV ≥ 3% feature who also presented with a 2–2.99% or 1–1.99% feature in the preceding year.

Cancer Site	Number of Participants with Feature ≥3% in 2016	Number (%, 95% Confidence Interval) of Participants with Feature ≥ 3% in 2016 Who Also Had a 2–2.99% Symptom in the Preceding Year		Number (%, 95% Confidence Interval) of Participants with Feature ≥ 3% in 2016 Who also Had a 1–1.99% Symptom in the Preceding Year	
		Number	% (95% CI)	Number	% (95% CI)
Bladder	252	0	0	15	6.0 (3.4 to 9.6)
Breast	321	0	0	5	1.6 (0.5 to 3.6)
Colorectal	5273	244	4.6 (4.1 to 5.2)	878	16.7 (15.7 to 17.7)
Endometrium	110	0	0	0	0
Kidney	263	0	0	15	5.7 (3.2 to 9.2)
Larynx	155	14	9.0 (5.0 to 14.7)	0	0
Lung	818	21	2.6 (1.6 to 3.9)	148	18.1 (15.5 to 20.9)
Ovary	160	8	5.0 (2.2 to 9.6)	0	0
Pancreas	78	0	0	2	2.6 (0.3 to 9.0)
Prostate	974	76	7.8 (6.2 to 9.7)	19	2.0 (1.2 to 3.0)
Oesophago-gastric	212	2	0.9 (0.1 to 3.4)	65	30.7 (24.5 to 37.3)
**Total**	**8616**	**365**	**4.2 (3.8 to 4.7)**	**1147**	**13.3 (12.6 to 14.0)**

## Data Availability

The anonymised participant data from this study are not available, in line with the CPRD’s data security policy. CPRD code libraries are available from the authors on request.

## Supplementary Materials

The following are available online at https://www.mdpi.com/article/10.3390/cancers15153936/s1, File S1: The supplementary excel file of Appendix A.

## Appendix A

Please see Supplementary Materials Excel file with individual sheets as follows:

**Sheet**	**Title**
S1	Information
S2	all sites summary
S3	all sites by time
S4	all features prevalence
S5	features prevalence 2–2.99
S6	features prevalence 1–1.99
S7	bladder summary
S8	bladder by time
S9	bladder by feature
S10	breast summary
S11	breast by time
S12	breast by feature
S13	colorectal summary
S14	colorectal by time
S15	colorectal by feature
S16	endometrium summary
S17	endometrium by time
S18	endometrium by feature
S19	kidney summary
S20	kidney by time
S21	kidney by feature
S22	larynx summary
S23	larynx by time
S24	larynx by feature
S25	lung summary
S26	lung by time
S27	lung by feature
S28	ovary summary
S29	ovary by time
S30	ovary by feature
S31	pancreas summary
S32	pancreas by time
S33	pancreas by feature
S34	prostate summary
S35	prostate by time
S36	prostate by feature
S37	upperGI summary
S38	upperGI by time
S39	upperGI by feature
